# Hydrolyzed Chicken Extract (ProBeptigen^®^) on Cognitive Function in Healthy Middle-Aged People: A Randomized Double-Blind Trial

**DOI:** 10.3390/nu12051362

**Published:** 2020-05-10

**Authors:** Dean Wu, Cheng-Chang Yang, Kuan-Yu Chen, Ying-Chin Lin, Pei-Jung Wu, Pei-Hsiu Hsieh, Yoshihiro Nakao, Mandy Y. L. Ow, Yi-Chen Hsieh, Chaur-Jong Hu

**Affiliations:** 1 Department of Neurology, Shuang-Ho Hospital, Taipei Medical University, New Taipei 235, Taiwan; 08242@s.tmu.edu.tw (D.W.); 19589@s.tmu.edu.tw (C.-C.Y.); 12163@s.tmu.edu.tw (K.-Y.C.); 13363@s.tmu.edu.tw (P.-J.W.); 16015@s.tmu.edu.tw (P.-H.H.); 2 School of Medicine, College of Medicine, Taipei Medical University, Taipei 110, Taiwan; green1990@tmu.edu.tw; 3 Research Center for Brain and Consciousness, Taipei Medical University, Taipei 235, Taiwan; 4 Department of Family Medicine, Wanfang Hospital, Taipei 116, Taiwan; 5 Research and Development, BRAND’S Suntory Asia, Singapore 138623, Singapore; Yoshihiro.Nakao@brands-suntory.com (Y.N.); Mandy.ow@brands-suntory.com (M.Y.L.O.); 6 PhD Program for Neural Regenerative Medicine, College of Medical Science and Technology, Taipei Medical University, Taipei 110, Taiwan

**Keywords:** dietary supplements, cognitive decline, essence of chicken

## Abstract

Cognitive decline is an important issue of global public health. Cognitive aging might begin at middle adulthood, the period particularly vulnerable to stress in lifespan. Essence of chicken (EOC) has consistently demonstrated its beneficial effects on various cognitive domains as nutritional supplementation. This study primarily aimed to examine the cognitive enhancement effects of ProBeptigen^®^ (previously named CMI-168), hydrolyzed peptides extracted from EOC, in healthy middle-aged people under mild stress. Ninety healthy subjects were randomly assigned into the ProBeptigen^®^ or placebo group for eight weeks. Neurocognitive assessment, event-related potentials (ERPs), and blood tests were conducted before, during, and after the treatment. The ProBeptigen^®^ group outperformed placebo group on Logical Memory subtests of Wechsler Memory Scale-third edition (WMS-III) and Spatial Working Memory task in the Cambridge Neuropsychological Test Automated Battery (CANTAB). The anti-inflammatory effects of ProBeptigen^®^ in humans were also confirmed, with progressively declining high-sensitivity C-reactive protein (hs-CRP) levels. Regular dietary supplementation of ProBeptigen^®^ is suggested to improve verbal short- and long-term memory as well as spatial working memory, and reduce inflammation in middle-aged healthy individuals with stress. The effects of ProBeptigen^®^ on cognition warrant further investigation. (NCT03612752)

## 1. Introduction

As the global population ages, cognitive decline and its terminal condition, dementia is emerging to be an important issue of public health in the world. It is generally agreed that cognitive decline may be a presentation of intricate interaction between multiple factors, such as demographics, genetics, physical and mental activities, socio-economic status, lifestyle and environmental factors [1]. Among these factors, aging may play one of the most important roles [2]. Cognitive aging representing a gradual decline in dedicated cognitive domains could begin nearly insidiously in middle age but accelerate in old age. Compelling evidence has supported that cognitive decline can be detected at middle adulthood, from the early 30s [3] to 45 [4] and 50 [5]. Another factor is inflammation, where greater systemic inflammation at midlife is also associated with steeper cognitive decline over the subsequent 20 years of life [6].

People at midlife are particularly vulnerable to stress, which also has a remarkable impact on cognitive functions, particularly attention and memory where the network of frontal–medial temporal regions is involved [7]. The perception of a scenario as overwhelming causes stress, which in turn elicits a measurable response resulting in a transformed status [8]. Although stress within moderate levels has been shown to improve job efficiency, excessive stress tends to induce negative consequences, such as anxiety, confusion, exhaustion, and burnout [9,10,11,12]. Existing evidence has proposed that stress-related neurocognitive decline is associated with low quality of life and a reduced adrenocorticotropic hormone (ACTH) in response to corticotrophin-releasing hormone [13].

Although this aforementioned cognitive decline may be still within a preclinical stage where formal medical intervention is not required, nutritional supplements are considered for cognitive enhancement protective or anti-cognitive aging. It is well-documented that particular nutrients have beneficial effects on cognition with scientific grounds, such as Mediterranean diets [14], antioxidants (lignans and vitamin E) [15], folate (itself or in conjunction with other B vitamins) [16], and other various minerals and micronutrients [17,18]. Recent studies have also confirmed that specific members of omega-3 polyunsaturated fatty acids (PUFA) may exert beneficial and neuroprotective effects on the aging brain [19] and cognitive functions [20,21].

Notably, protein and peptides have been identified as the key nutrients for improving reaction time in elderly [22] and cognitive performance in stress-vulnerable subjects [23]. With regard to dietary supplements of protein and peptides, essence of chicken (EOC) should be highlighted with its ingredients of rich proteins and peptides, trace elements, carnosine, anserine, and amino acids with very low fat. EOC is a liquid form of highly-concentrated extracts from cooked chicken meat which has been regarded as valuable nourishment with a long history of consumption in Eastern Asia. Studies on EOC have consistently demonstrated its beneficial effects on various cognitive domains, such as working memory and attention [24,25,26]. In our prior study, participants with high worked-related stress and concomitant depressive mood showed a significant improvement in short-term memory after regular EOC consumption for two weeks [27]. Utilizing a proprietary bio-processing technology, EOC has been further enriched from EOC via hydrolysis to produce ProBeptigen.

ProBeptigen^®^, previously named “CMI-168”, is a chicken meat hydrolysate comprised of chicken peptides and amino acids. The safety and toxicity of ProBeptigen^®^ have previously been investigated in two animal studies [28,29] and one unpublished clinical trial [30]. In one animal study [28], ProBeptigen^®^ was orally administered at three doses of 500, 1000, and 2000 mg/kg/day for 28 days in Sprague-Dawley rats. The group with the high dose (2000 mg/kg/day) was observed for another 14 days after stopping administration of ProBeptigen^®^. The study results showed no toxicity in all groups, in term of observation of activities, body weight, food and water intake, and examinations of pathology (hematology, blood chemistry, and urinalysis, organ weights, and histopathological examination). In another higher-dose 28-day toxicity study [29], two doses of 6 and 12 g/kg/day of ProBeptigen^®^ were administered to Sprague-Dawley rats. Neither toxicity nor adverse effects were observed in these rats subjected to extremely high doses of ProBeptigen^®^. In the clinical study [30], 25 healthy subjects consumed high-dose (8 tablets/day) ProBeptigen^®^ (335 mg/tablet) for four weeks while another group of 25 healthy subjects with body mass index (BMI) ≥ 24 consumed the usual dose (two tablets/day) for 12 weeks. Laboratory (blood biochemistry, hematology, liver function, and renal function including acute kidney injury biomarkers, and urinalysis) markers and adverse events reported during consumption and two weeks after stopping the supplementation were not significantly different between placebo and ProBeptigen^®^ groups.

With respect to the cognitive enhancement of ProBeptigen^®^, three animals [31,32] and one human studies [33] have been conducted. In middle-aged mice (9–12 months old), daily intake of ProBeptigen^®^ 150 mg/kg of body weight for six weeks improved the hippocampus-related non-spatial memory measured by novel object recognition test (NOR), independent of structural or functional changes in the hippocampus. ProBeptigen^®^ was also found to protect the mice against stress-induced weight loss in that study [31]. In the senescence-accelerated prone (SAMP8) mice study, oral consumption of ProBeptigen^®^ at three different doses (150, 300, or 600 mg/kg/day) for 13 weeks reduced the scores of senescence and improved learning and memory. ProBeptigen^®^ supplementation also increased antioxidant enzyme activity and dopamine level. It reduced protein and lipid peroxidation and mitochondrial DNA damage in the brain. Brain dopamine, which is reduced in aged SAMP8 mice, was increased in the ProBeptigen^®^ group. Attenuating the decrease in dopamine may improve cognition and help in coping of acute and chronic stress, as dopamine is a neurotransmitter of the sympathetic nervous system. The microarray analysis of the hippocampus revealed changes in several gene expressions that may be involved in the improvement of cognition by ProBeptigen^®^. These results imply a potential anti-aging effect of ProBeptigen^®^ in alleviating cognitive deficits and promoting the antioxidant defense system [32]. In the most recent study using naturally aged mice, ProBeptigen^®^ ameliorated age-induced cognitive decline, reduced oxidative stress, and reduced neuroinflammation in the hippocampus and cortex [34]. A pilot clinical study [33] on 20 healthy subjects with six weeks of ProBeptigen^®^ (670 mg/day) consumption showed that ProBeptigen^®^ enhanced cognitive performances on Digit Span backwards, Letter–Number Sequencing, and the Rey Auditory Verbal Learning Test (RAVLT), in terms of working memory and episodic memory functions. Notably, the enhancement persisted for two weeks after stopping the treatment and none of the participants reported adverse effects during or after the treatment.

Based on the aforementioned empirical findings, further investigations concerning the effectiveness of ProBeptigen^®^ on cognitive function in healthy middle-aged people under mild stress are warranted, particularly implemented with a larger sample size and comprehensive cognitive evaluations. The present study primarily aimed to examine the cognitive enhancement effects from ProBeptigen^®^ in healthy middle-aged people under mild stress. Factors that may compromise neurocognitive function, such as mood and sleep quality, were also assessed as secondary outcomes. Meanwhile, blood biochemistry data were collected for the effects of ProBeptigen^®^ on physical conditions and safety monitoring.

## 2. Materials and Methods

### 2.1. Study Design and Participants

**Figure 1:**
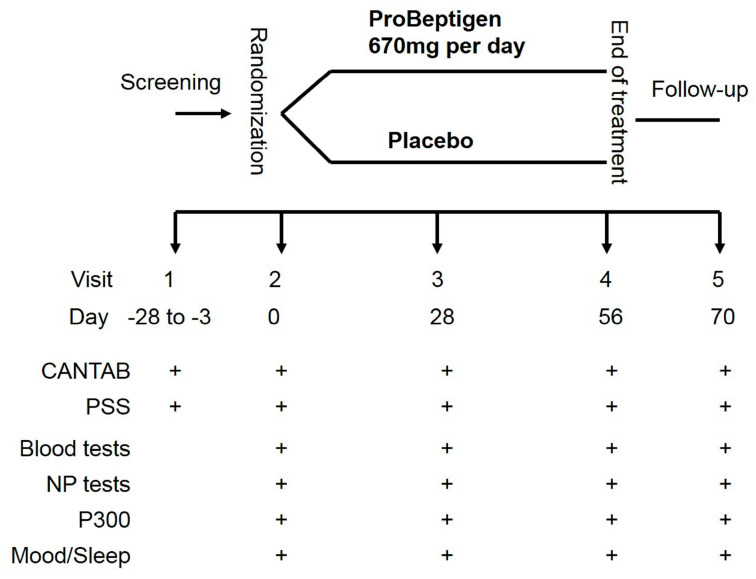
The study protocol. CANTAB, Cambridge Neuropsychological Test Automated Battery; The symbol of “+” denotes the examination performed at that visit. NP, neuropsychological; PSS, Perceived Stress Scale; P300, event-related potential.

This study was a randomized, double-blind, parallel placebo-controlled trial. The participants and investigators were both blinded to the participant allocation. Participants who met the inclusion criteria were stratified by sex and randomly assigned into the ProBeptigen^®^ or placebo group for eight weeks after enrollment (Figure 1). A computer-generated randomization list was provided by the sponsor to the study site. At the study site, subjects were sequentially assigned to the next randomization number. The inclusion criteria were: (1) healthy adults aged between 35–65 years old with normal BMI from 18.0 to 30.0 kg/m^2^ and above 40 kg in bodyweight at screening; (2) ability to understand and write Traditional Chinese or at least have completed primary school education; (3) normal performances measured by Cambridge Neuropsychological Test Automated Battery (CANTAB) [35]; and (4) a mild stress level, defined by the score on Perceived Stress Scale (PSS) [36] over 20. The exclusion criteria were: (1) existing or previous psychiatric diseases (bipolar disorder and schizophrenia), neurological diseases, dementia, severe head injury, cerebrovascular diseases, Parkinson’s disease, or other systemic diseases, including respiratory diseases, cancers, and diabetes; (2) the abstinence time of any psychotropic medication, such as anxiolytics and sedatives less than three months; (3) a history of allergy to chicken meat or other protein-based food or supplements; (4) long-term consumption of dietary supplement or herbal products likely to have an effect on memory; (5) smoking more than 10 cigarettes per day; or (6) alcoholism.

### 2.2. Study Procedure

As per Figure 1, the screening evaluation was completed in visit 1 (3–28 days before the first treatment). All the participants gave the written informed consent before the study commencement. The participants took either 2 tablets (335 mg/tablet) of ProBeptigen^®^ or placebo once daily in the morning, depending on their random allocation. Neurocognitive assessment, event-related potentials (ERPs) indexed by the P300 component, and blood tests were performed at visit 2 (baseline, before treatment), visit 3 (28 days after treatment commencement), visit 4 (56 days after treatment commencement) and visit 5 (two weeks post-treatment termination). For special interests on the potential effects of ProBeptigen^®^ on mood and sleep quality, Beck Depression Inventory-second edition (BDI-II) [37], State-Trait Anxiety Inventory (STAI) [38], and Pittsburg Sleep Quality Index (PSQI) [39] were also performed on the same aforementioned occasions. The primary outcome was the change of cognitive function from baseline in at least one of the following visits between the placebo and treatment groups. The secondary outcomes were the differences of those indicator changes after treatment on the blood tests, P300, mood, and sleep quality between groups.

### 2.3. ProBeptigen^®^ and Placebo

ProBeptigen^®^ is a hydrolyzed chicken extract prepared from chicken meat which has undergone proprietary bio-processing technology and aqueous extraction. It comprises 91.4% protein (4.2% free amino acids, 7.6% diketopiperazines or cyclic dipeptides), 1.0% carbohydrate, 1.6% lipid, 2.0% moisture, and 4.0% minerals and ash. Specifically, diketopiperazines and their derivatives have shown neuroprotective or nootropic effects [40,41]. Two of them found in ProBeptigen^®^ are cyclo-prolylglycine (cyclo-(Pro-Gly)) and cyclo(L-Phe-L-Phe), which have exhibited anti-amnesic activity [42,43]. Three pro-cognition amino acids are also identified in ProBeptigen^®^, including glutamate, arginine, and tyrosine, which consequently contribute to recognition memory enhancement [44], vasodilatation and neuroprotective effects [45,46,47], and working memory improvement [48,49,50], respectively.

Participants were requested to take two tablets of ProBeptigen^®^ (335 mg/tablet, manufactured by BRAND’S Suntory Asia, Singapore, Singapore) or placebo (335 mg/tablet, manufactured by BRAND’S Suntory Asia) after meals every morning consecutively for eight weeks (56 days). The placebo tablet was identical to ProBeptigen^®^ in appearance, made of microcrystalline cellulose.

### 2.4. Measurements

#### 2.4.1. Neurocognitive Tests

Licensed clinical neuropsychologists blinded to the participant allocation administered the neuropsychological assessments. These neurocognitive instruments included the Logical Memory and Family Pictures subtests of the Wechsler Memory Scale-3rd edition (WMS-III) [51] and CANTAB.

The WMS-III is one of the most widespread memory assessments consisting of 10 primary and seven optional subtests. The Logical Memory subtests require the examinee to remember narrative stories presented orally. The Logical Memory I and II assess immediate and subsequent delayed recall, respectively. The Family Pictures subtests require the examinee to remember information of four colored pictures regarding seven family members acting in four different scenes visually presented in a sequential fashion. Similarly, the Family Pictures I and II examine immediate and delayed spatial memory respectively. The raw score of each index obtained from each subtest is transferred into a standardized score (mean = 10, standard deviation = 3) [52]. The Taiwanese version of WMS-III was used in the present study [53].

The CANTAB is one of the most extensively used computerized test batteries used to assess cognition via a tablet with a touch screen [54,55]. The CANTAB tasks are considered language independent and non-sensitive to gender with satisfactory levels of reliability and validity [56]. Given that a variety of CANTAB tasks were selected in the current study, detailed information regarding these tasks was described elsewhere (see the online Supplementary Material Tables S1 and S2).

#### 2.4.2. Self-Reported Instruments

The PSS is comprised of 14 items on a 5-point Likert scale (from 0 to 4) measuring the respondent’s subjective level of stress in the daily community situations, with higher scores indicating greater perceived stress. Given that no official cut-off score is available, the current study used a score of 20 as the criterion considering the means of the PSS in the previous study [57] falling around 17 to 19.

The BDI-II [37] consists of 21 self-rating items scored on a 4-point Likert scale (from 0 to 3) with a total score ranging from 0 to 63. In accordance with the BDI-II manual, the severity of depression can be distinguished by the total score as normal (0–13), mild (14–19), moderate (20–28), or severe (29–63). The traditional Chinese version of BDI-II was used in the study with the high internal consistency (Cronbach’s α = 0.94) [58] and test–retest reliability of 0.86 [59].

The STAI is one of the psychometrically validated instruments aiming to assess both state and trait anxiety separately [38]. Each type of anxiety consists of 20 different self-reported questions on a 4-point Likert scale (from 1 to 4); thus, scores in each subscale range from 20 to 80, with higher scores correlating with greater anxiety. The Taiwanese version of STAI used here has been shown good psychometrical properties [60].

Overall sleep quality was assessed using the PSQI [39]. It consists of 19 self-reported items contributing to seven components of sleep quality. A high overall score on represents a poor sleep quality with a clinical cut-off score of 5. The Taiwanese version of the PSQI has been validated and used in the current study [61] (see the online Supplementary Material Table S3 for the reference values of self-report instruments).

#### 2.4.3. Blood Tests

Several biochemistry markers were examined at visit 2–5, including liver function: represented by Alanine transaminase (ALT) and aspartate aminotransferase (AST); renal function: indexed by blood urea nitrogen (BUN), creatinine (CR), and estimated glomerular filtration rate (eGFR); thyroid function: indexed by triiodothyronine (T3), thyroxine (T4), and thyroid-stimulating hormone (TSH); physiological index of stress: cortisol at 8 am; inflammation indicator: high-sensitivity C-reactive protein (hs-CRP); and the fasting glucose level (see the online Supplementary Material Table S4 for the reference range).

#### 2.4.4. ERPs

ERPs are very small voltages recorded from the scalp electroencephalography (EEG) originated from brain cortex in response to specific events or stimuli. The P300 is characterized by a large positive elicited wave which peaks around 300 ms after target stimulus onset in the process of decision-making; prominent over the parietal region [62]. The auditory P300 ERP appears when the patient is presented with an incongruent stimulus unexpectedly during a stimulus discrimination task. The P300 wave is analyzed by the size of the deflection (amplitude) and the time elapsed post-stimulus before activation (latency). Conventionally, shorter P300 latencies and larger amplitudes are associated with superior information processing in the brain [63,64]. In this study, auditory P300 was used as a physiological indicator for subjective cognitive functions. For the auditory ERP paradigm, binaural 750 (standard) and 2000 (target) Hz tones were delivered through headphones at 75 dB. The two kinds of tones were presented in random sequence once every 1 s, with the constraint that target stimulus probability was 0.20 in 360 trials. Brain activities were recorded at the Fz and Cz electrode sites with ears (A1, A2) as references. Both the amplitude and latency at the Cz were collected and analyzed. The setting was according to the routine clinical examinations.

### 2.5. Statistical Analysis

Sample size calculations indicated that a total sample size of 72 subjects would be needed to observe the anticipated effect size f of 0.3 with a power of 0.80 and a correlation of 0.70 between repeated measures under repeated measures ANOVA methods using G*Power 3.1 software [65] according to a previous pilot study examining the effects of the ProBeptigen^®^ on cognition in healthy adults. Demographic characteristics, neurocognitive function, and biochemistry profiles at baseline were compared between the ProBeptigen^®^ and placebo groups using the independent Student’s *t*-test for continuous variables and the chi-square (χ²) test for categorical variables. Assessment of the change for neurocognitive function and biochemistry profiles between two groups from visit 2 to visit 5 were analyzed by applying generalized estimating equations (GEE) approach [66] with unstructured matrix model, which is commonly used for the analysis of longitudinal correlated data to obtain unbiased estimates of coefficients. The *p*-value obtained was the interaction term of treatment and time after considering the baseline data as a covariate, suggesting the result between the ProBeptigen^®^ and placebo groups changed over time. All statistical analyses were performed using SAS Version 9.4 (SAS Institute Inc., Cary, NC, USA) considering two-sided probabilities with a *p*-value < 0.05.

### 2.6. Ethical Approval and Trial Registration

This study was conducted in accordance with the Declaration of Helsinki and approved by the Joint Institutional Review Board of Taipei Medical University (reference no. N201711060). The study was also registered in ClinicalTrials.gov with the registration number NCT03612752.

## 3. Results

According to Section 2.5, a significant difference in the change gradient between the two groups was interpreted as the significant interaction with a covariate using the baseline data, while the individual post-hoc test comparing the two groups at each visit were not significant if not addressed specifically.

### 3.1. Participant Charateristics

**Figure 2:**
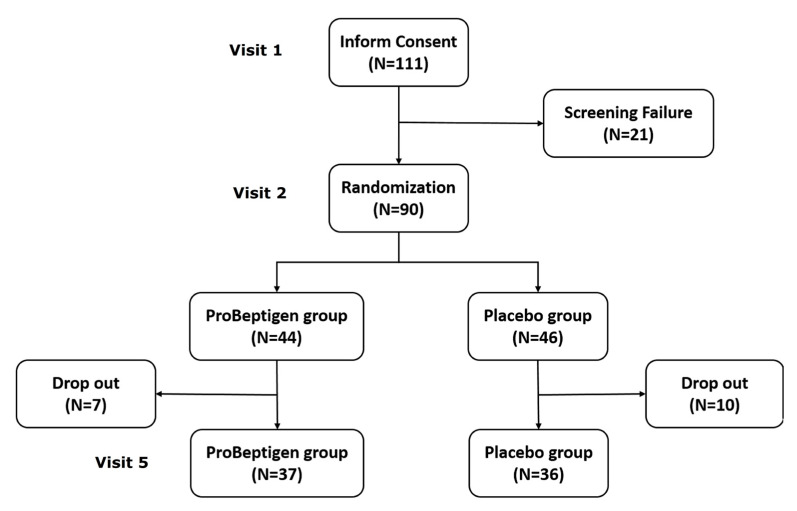
The flowchart of this clinical trial.

One hundred and eleven adults were initially recruited but 90 of them (81.1%) were considered eligible for the subsequent randomization (Figure 2). The major reason of ineligibility was the subnormal performance on CANTAB. A total of 73 participants completed the trial with compatible drop-out rates of 16.3% and 21.3% in ProBeptigen^®^ and placebo groups, respectively (χ² = 0.499, *p* = 0.480). No significant adverse events were reported and none of the drop-outs left because of intolerance of ProBeptigen^®^ or placebo. The drop-out reasons were other commitments during the follow-up visits (12/17), pregnancy (1/17), technical errors in CANTAB at baseline (1/17), having influenza (1/17), and unknown causes (2/17). The drop-out rate is considered acceptable in the context of a non-life-threatening scenario for eight weeks.

There were no differences between groups on baseline demographics (age, education year), neurocognitive tests, perceived stress, ERPs, and plasma biochemistry profile, except for the fasting glucose level (Table 1).

### 3.2. Neurocognitive Outcomes

#### 3.2.1. WMS-III

**Figure 3:**
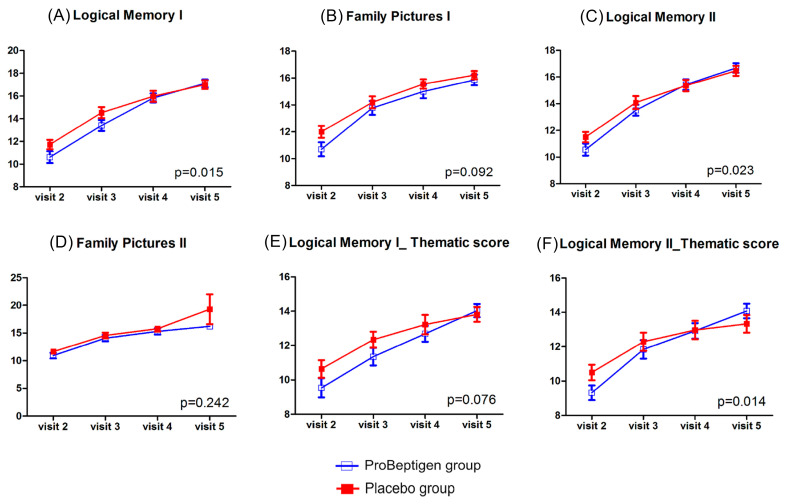
Results from Logical Memory and Family Pictures subtests of the Wechsler Memory Scale-3rd edition. (**A**) Logical Memory I, (**B**) Family Pictures I, (**C**) Logical Memory II, (**D**) Family Pictures II, (E) Thematic score of Logical Memory I, (F) Thematic score of Logical Memory II. The unit of the Y-axis is scaled score. Error bars represent standard errors. The *p*-value shown in each diagram denotes the statistics of time × treatment interaction after considering the baseline data as a covariate. Higher scores indicate better performance.

There was a significant time × treatment interaction (*p* = 0.015) for the Logical Memory I subtest, suggesting there was more improvement of verbal immediate recall in the ProBeptigen^®^ group (mean score difference from visit 2 to visit 5, 6.49 ± 0.51) than that in the placebo group (mean score difference from visit 2 to visit 5, 5.28 ± 0.37). Similarly, there was a significant time × treatment interaction (*p* = 0.023) for the Logical Memory II subtest, implying a much better progress of verbal long-term recall for the ProBeptigen^®^ group (mean score difference from visit 2 to visit 5, 6.11 ± 0.50) than the placebo group (mean score difference from visit 2 to visit 5, 4.97 ± 0.32). In addition, the ProBeptigen^®^ group had a greater long-term gist memory (mean score difference from visit 2 to visit 5, 4.75 ± 0.51) than the placebo group (mean score difference from visit 2 to visit 5, 2.83 ± 0.49), reflected by a significant time × treatment interaction for the Logical Memory II thematic score (*p* = 0.014). There were no significant interaction effects for the Logical Memory I thematic score, Family Pictures I subtest, and Family Pictures II subtest (Figure 3).

#### 3.2.2. CANTAB

**Figure 4:**
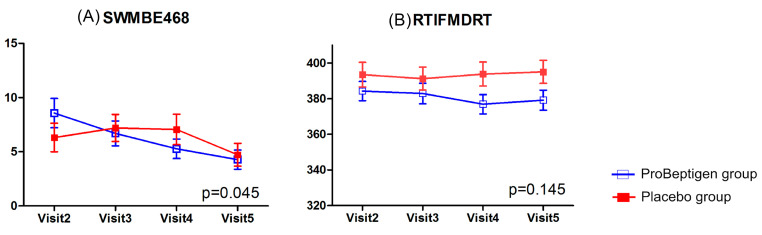
CANTAB including (**A**) spatial working memory (SWMBE468) and (**B**) reaction time task median five-choice reaction time (RTIFMDRT) results. Error bars represent standard errors. The *p*-value shown in each diagram denotes the statistics of time × treatment interaction after considering the baseline data as a covariate. Lower scores indicate better performance.

The changes of spatial working memory (SWMBE468) from visit 2 to visit 5 suggested that significant improvements (*p* = 0.045) in performance were observed in the ProBeptigen^®^ group (mean score difference from visit 2 to visit 5, −4.29 ± 1.31) when compared to placebo group (mean score difference from visit 2 to visit 5, −1.58 ± 0.97). There was no significant time × treatment interaction (*p* = 0.145) in psychomotor speed performance (RTIFMDRT). No performances on other tasks of CANTAB showed significant interaction effects (Figure 4).

### 3.3. Self-reported Measurements

**Figure 5:**
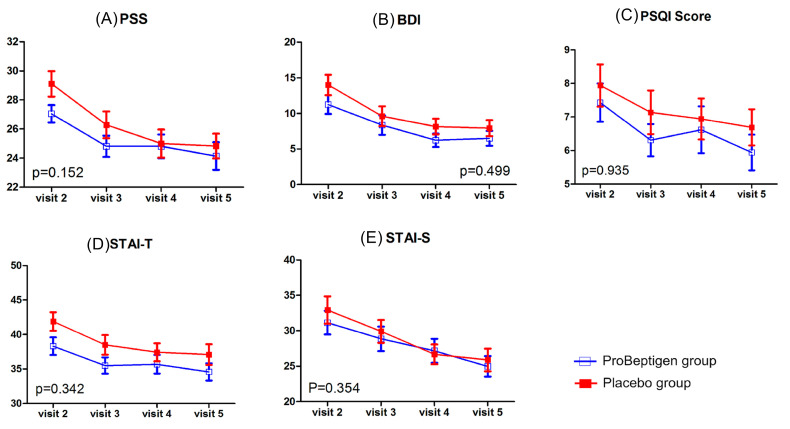
Results of perceived stress, depression, anxiety and sleep quality measurements. (**A**) Perceived Stress Score (PSS), (**B**) Beck Depression Inventory-II (BDI), (**C**) Pittsburg Sleep Quality Index (PSQI), (**D**) State-Trait Anxiety Inventory–trait anxiety (STAI-T), (**E**) STAI-S, State-Trait Anxiety Inventory–state anxiety (STAI-S). Error bars represent standard errors. The *p*-value shown in each diagram denotes the statistics of time × treatment interaction after considering the baseline data as a covariate. Lower scores indicate fewer symptoms.

There was no significant time × treatment interaction in PSS (*p* = 0.152), BDI-II (*p* = 0.499), the state anxiety subdomain (*p* = 0.354) and trait anxiety subdomain (*p* = 0.342) of STAI, and PSQI (*p* = 0.935; Figure 5).

### 3.4. Physiological Outcomes

**Figure 6:**
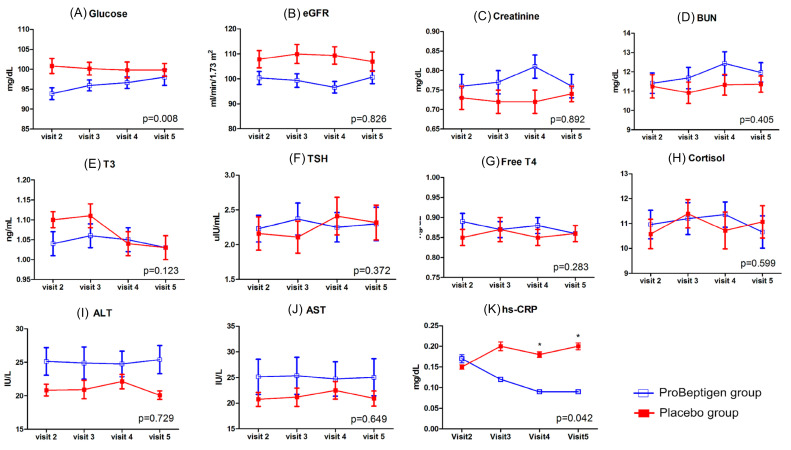
Changes of biochemistry profiles. (**A**) Glucose, (**B**) Estimated glomerular filtration rate (eGFR), (**C**) Creatinine, (**D**) Blood urea nitrogen (BUN), (**E**) Triiodothyronine (T3), (**F**) Thyroid-stimulating hormone (TSH), (**G**) Free thyroxine (Free T4), (**H**) Cortisol, (**I**) Alanine transaminase (ALT), (**J**) Aspartate aminotransferase (AST), (**K**) High-sensitivity C-reactive protein (hs-CRP). Error bars represent standard errors. The *p*-value shown in each diagram denotes the statistics of time × treatment interaction after considering the baseline data as a covariate and the asterisk denotes a statistically significant between-group difference at the specific visit time.

**Figure 7:**
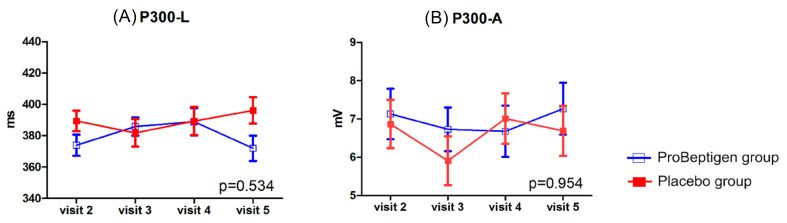
Results of event-related potentials. (**A**) Latency of positive event-related potentials at 300 ms (P300-L), (**B**) Amplitude of positive event-related potentials at 300 ms (P300-A). Error bars represent standard errors. The *p*-value shown in each diagram denotes the statistics of time × treatment interaction after considering the baseline data as a covariate.

There was increase of plasma fasting glucose levels in ProBeptigen^®^ group when compared to placebo group (*p* = 0.008) (Figure 6). One outlier was identified in ProBeptigen^®^ group and the follow-up examination of fasting blood sugar on this participant was within normal range (data not shown). The inflammation indicator, hs-CRP showed a statistically significant result (*p* = 0.042), suggesting that ProBeptigen^®^ group had decreased hs-CRP level over time (mean score difference from visit 2 to visit 5, −0.08 ± 0.05) when compared to placebo group (mean score difference from visit 2 to visit 5, 0.04 ± 0.05). Furthermore, the pairwise comparison showed a significant difference between two groups at visit 4 and visit 5. The results of P300 did not show significant interaction effects in latency and amplitude between two groups (Figure 7).

## 4. Discussion

The primary goal of the current study aims to evaluate the efficacy of ProBeptigen^®^ in improving cognitive function using a randomized, double-blind, parallel placebo-controlled study design. Given that there was only one pilot trial investigating the relevant issue, the current study provides incremental evidence concerning the cognitive effects of ProBeptigen^®^. Subjects consuming 670 mg ProBeptigen^®^ daily for eight weeks improved their visual working memory and verbal memory performance compared to the placebo group. Similarly, inflammation measured with hs-CRP, also declined with time in the ProBeptigen^®^ group and remained low two weeks after stopping supplementation. Selective cognitive enhancement effects of ProBeptigen^®^ were identified while the test modality (auditory vs. visual) appeared to play a crucial role in the results of the current study.

The results of the current study are largely in line with the prior animal [31,32] and human studies [33]. In one study using middle-aged mice investigating the cognitive effectiveness of ProBeptigen^®^ on spatial and non-spatial memory [30], only a material-specific memory (non-spatial) improvement was found. The other animal study [31] and pilot trial in humans [32] not manipulating the modality of memory tasks also revealed improvements in memory and learning after ProBeptigen^®^ consumption. Taken together, the results of the current and previous research suggest that ProBeptigen^®^ has material-specific beneficial effects in non-spatial memory. It is noteworthy that the not only verbatim memory but also gist-based memory (measured by the thematic score of Logical Memory subtests) are improved in ProBeptigen^®^ group. This result highlights the potential beneficial effects of ProBeptigen^®^ in memory, particularly for older adults as they tend to more rely on gist-based memory (a general meaning or idea conveyed by an assortment of items) [67]; however, relevant research dedicated for geriatric population is still required.

With cognitive domains other than memory, it is interesting that only improved spatial working memory was noted in the ProBeptigen^®^ group. Given that no auditory or verbal working memory tasks were administered to these participants, it is not possible to address the material-specific effects of ProBeptigen^®^ on working memory so far. Although enhanced auditory/verbal working memory effects from ProBeptigen^®^ or other EOC in healthy adults have been argued in one recent meta-analysis [68], it is still premature to draw a firm conclusion that ProBeptigen^®^ may improve both visual and verbal working memory. It is well known that working memory capacity persistently declines with aging [69]; therefore, such a cognitive facilitation effect in working memory should be appealing to the middle-aged and elderly. In the classical model of working memory, working memory refers to a limited capacity system allowing the temporary storage (i.e., memory) and manipulation of information (pertaining to executive function and processing speed) necessary for complex tasks [70]. It is not clear that the improved spatial working memory in the current study is a standalone phenomenon or is secondary to improved memory (less likely since visual memory is not improved significantly), executive function (not measured in the study), or processing speed (improvement manifested on the last occasion), since such a differentiation is beyond the scope of the current study. In addition, since visuospatial function may mediate visuospatial (working) memory, this confounder is worthy to be taken into account in the future. On the other hand, psychomotor (processing) speed is often assumed to be the core issue responsible for defective cognitive performances in the elderly [71]. As the current study revealed a potential benefit of ProBeptigen^®^ on psychomotor speed particularly at later visits, future studies of a longer duration could continue the investigation of ProBeptigen^®^ on psychomotor speed, which may in turn contribute to various other cognitive domains.

This study purposefully included middle-aged subjects due to mechanisms of action of ProBeptigen^®^ beneficial in aging, and preclinical evidence of learning and memory efficacy in middle-aged or aged mice. Specifically, the antioxidant [32] and anti-inflammatory [34] mechanisms, reduction of mitochondrial DNA damage in the brain, and increased dopamine levels [32] are plausible mechanisms of action for improving cognitive function in middle-aged subjects. In this trial, the anti-inflammatory effects of ProBeptigen^®^ was confirmed in humans, as hs-CRP levels declined over time. The real causes why ProBeptigen^®^ improved mainly the auditory–verbal long-term memory certainly need further investigation. Another important finding requiring further investigation is that the beneficial cognitive effects of ProBeptigen^®^ persisted two weeks after the treatment termination. It suggests that ProBeptigen^®^ with longer-term supplementation has further potential to retain its cognitive enhancement effects for a longer duration; however, the period of the after-effects needs further investigation with extended and multiple follow-ups. Furthermore, the effect of ProBeptigen^®^ in cognitive enhancement may be a result of consumption of aforementioned specific amino acids or peptides of its ingredients; however, the exact mechanism and nature of the potential bioactive(s) would require further investigation.

Given that the previous pilot study regarding this issue did not take confounding variables, such as stress, mood, and sleep quality into consideration, this study recruited the participants with stress and monitored the progression of stress, anxiety, depression, and sleep quality. Although all these confounders decreased with time progression in both groups, no between group difference was found. Therefore, ProBeptigen^®^ is assumed to have no negative impacts on stress, anxiety, depression, and sleep quality and the cognitive improvements found in ProBeptigen^®^ group should be free from these personal characteristics.

Another important attempt made in the current study was to index an objective measurement in cognition using the P300 components of ERPs. The use of P300 as a measurement of cognitive deterioration in Alzheimer’s disease (AD) pathology has been characterized for several decades since Goodin, Squires, and Starr first reported the clinical utility of the auditory P300 latency in patients with dementia in 1978 [72]. The P300 is then considered a specific and sensitive measure of afferent function in neurological disorders, especially for detecting progressive cognitive decline with the prolongation of the P300 latency in AD. In our study, the P300 latency and amplitude showed no significant differences in all visits between groups, while trends of a shorter latency and higher amplitude were identified in ProBeptigen^®^ group at the last visit. It is possible that the eight-week supplementation in the current study is not sufficient to reveal significant differences; therefore, a longer period of ProBeptigen^®^ consumption might be proposed. Moreover, it is not surprising that negative findings from P300 components were found in the current study, as P300 represents an early attentional process requiring low level of demand in cognitive reservoirs [64,73], which should be intact in our healthy participants. Since alternative indicators of cognition evaluations should be required apart from neuropsychological assessments only, future studies may consider including more neurophysiological measurements for cognition monitoring.

The blood biochemistry examination along the treatment period did not show changes before and after treatment in both groups, except for increased mean fasting glucose levels in ProBeptigen^®^ group at the follow-up (two weeks after the treatment termination), with one outlier identified and a normal fasting glucose on follow-up examination. This incident is unexpectedly contrary to findings in prior animal and human studies using dietary supplements of ProBeptigen^®^ [28,29,30] or EOC [74]. Therefore, it is prudent to monitor fasting blood sugar regularly in future ProBeptigen^®^ clinical trials to confirm its safety. The declining levels of hs-CRP, an indicator of inflammation and cardiovascular risks, during and after ProBeptigen^®^ treatment is noteworthy. Chronic low-grade inflammation is a hallmark of aging, and a recent study [34] in naturally aged mice showed similarly that ProBeptigen^®^ exerted anti-neuroinflammatory effects and significantly diminished levels of pro-inflammatory cytokines, tumor necrosis factor alpha (TNF-α), and interleukin-1 beta (IL-1β), in the hippocampus and cortex, alongside antioxidant effects and improved learning and memory. It is suggested that the reduced levels of these cytokines could be explained by significantly decreased phosphorylation levels of nuclear factor-kB (NF-kB) p65 which may contribute to many age-related neuroinflammatory diseases if immunoreactivity is enhanced for NF-kB signaling [75]. This trial confirmed the anti-inflammatory effects in humans, which could in part explain the enhanced cognitive function over time observed in the ProBeptigen^®^ group.

The current study is not free from limitations. A potential selection bias arises from the considerable number of candidates excluded at screening due to subnormal performances on CANTAB. Although CANTAB has been used worldwide, the nature of computerized administration may still prevent some individuals with normal cognition who are not familiar with high-tech products from performing at the expected level [76]. Given that the performance distributions of enrolled participants on WMS-III at baseline are compatible with those of the normative population (i.e., mean ± standard deviation = 10 ± 3), enrolled participants should be representative of the normal population. One may also contend that criterion of mild stress may reduce the generalizability of the current findings to general populations. However, the cut-off of 20 was defined arbitrarily while the distribution score of PSS in Chinese community population is 22.42 ± 5.74 [77]. Considering the score distribution of PSS at the commencement of the current study, at least a half of participants whose PSS scores were within 1 standard deviation above the mean. In addition, given that PSS is a brief self-appraisal of daily life stress, it is not a diagnostic measure of psychological symptomatology [36]. Therefore, the participants should be viewed as normal healthy adults with higher perceived daily life stress while the partial generalizability of the findings in the current study should be mentioned. Appraised stress may be symptomatic of psychological disorder when viewed in combination with elevated scores on other psychiatric symptoms, it is our contention that the perception of stress itself, as assessed by the PSS.

Another potential limitation could have arisen due to a slight imbalance (albeit not significant) in baseline cognitive function between groups in some domains, arising by chance from random allocation. Since there were subtle differences at baseline, cognitive performance may have improved more due to lower scores at baseline in the active group, as lower scores could indicate more room for improvement. Nevertheless, in another test (Family Pictures I subtest) where the ProBeptigen^®^ group similarly performed poorer at baseline, ProBeptigen^®^ did not improve more than placebo did. This suggests that while some effects may be due to a greater room for improvement at baseline, a large enough treatment effect still needs to be present in order to achieve a significantly different improvement rate. Also, in our study, apart from the results of hs-CRP, only significant interactions were detected with no presence of statistically significant post-hoc tests. This implies that the rate of improvement was different; however, the two groups did not perform differently at any time point. One reason could be the presence of subtle baseline imbalances as discussed above. As could be illustrated best in Figure 3 with the Logical Memory II thematic score, starting off at a lower (albeit not significant) baseline also makes it more difficult to show difference at any time point. Another possibility is that while greater improvement rate was seen through to eight weeks, the effect size was not large enough to show difference at a time point up to eight weeks of supplementation. To directly address the limitations of this trial, future trials could take proactive approaches to ensure balanced randomization of cognitive function at baseline, such as with minimization techniques, and to prolong the consumption period beyond eight weeks.

Other limitations, such as no cognitive evaluation of executive or visual–spatial function included in the present study should also be addressed for future studies attempting to comprehensively examine the effects of ProBeptigen^®^ on neurocognitive functions. Finally, a relatively small number of participants and brief time of treatment may be considered as the methodological weakness. Future studies should consider recruiting the elderly population for their higher incentives to adhere to a long-term treatment regimen concerning health promotion and disease prevention.

## 5. Conclusions

In conclusion, daily ProBeptigen^®^ 670 mg consumption for two months is suggested to improve verbal short- and long-term memory, as well as spatial working memory in middle-aged healthy individuals with perceived stress. Its anti-inflammatory effect was confirmed in humans, which may in part contribute to cognitive enhancement. Generally, ProBeptigen^®^ has no detrimental effects on other cognitive domains, stress, mood, sleep quality, and biochemical markers. Indicators showing a trend of changes, such as reaction time task are encouraged to be included in future studies to clarify ProBeptigen^®^ effects on these outcomes. The potential benefit of this supplementation on psychomotor speed should be verified in future studies.

## Figures and Tables

**Table 1:** Baseline (visit 2) information of participants who completed the whole study.

Variables ^1^	Total (*N* = 73)	ProBeptigen^®^ (*n* = 37)	Placebo (*n* = 36)	*p*-Value
Demographics				
Age (year)	42.38 ± 6.36	42.46 ± 6.36	42.31 ± 6.45	0.919
Education (year)	15.82 ± 1.77	15.62 ± 1.92	16.03 ± 1.59	0.328
WMS-III ^2^				
Logical Memory I	11.16 ± 2.85	10.62 ± 3.14	11.72 ± 2.44	0.100
Logical Memory I thematic score	10.08 ± 3.27	9.54 ± 3.42	10.64 ± 3.05	0.153
Logical Memory II	11.03 ± 2.59	10.57 ± 2.75	11.50 ± 2.36	0.125
Logical Memory II-thematic score	9.90 ± 2.72	9.32 ± 2.64	10.50 ± 2.72	0.065
Family Pictures I	11.34 ± 3.00	10.70 ± 3.15	12.00 ± 2.72	0.064
Family Pictures II	11.27 ± 2.96	10.92 ± 3.09	11.64 ± 2.83	0.308
Self-reported measures				
PSS	28.07 ± 4.59	27.05 ± 3.64	29.11 ± 5.25	0.057
BDI	12.60 ± 8.41	11.24 ± 8.06	14.00 ± 8.64	0.163
STAI-State	32.01 ± 10.84	31.14 ± 10.15	32.92 ± 11.59	0.487
STAI-Trait	40.07 ± 8.04	38.30 ± 7.76	41.89 ± 8.03	0.056
PSQI	7.68 ± 3.63	7.43 ± 3.48	7.94 ± 3.81	0.550
Event-related potentials				
P300-L (ms)	381.60 ± 40.79	373.95 ± 41.10	389.47 ± 39.49	0.104
P300-A (mV)	7.00 ± 3.87	7.13 ± 3.99	6.87 ± 3.80	0.781
Blood biochemistry				
ALT (IU/L)	22.97 ± 15.96	25.14 ± 20.86	20.75 ± 8.13	0.240
AST (IU/L)	23.03 ± 9.79	25.14 ± 12.47	20.86 ± 5.25	0.061
BUN (mg/dL)	11.33 ± 3.40	11.41 ± 3.24	11.25 ± 3.60	0.847
Cortisol (μg/dL)	10.77 ± 3.51	10.96 ± 3.49	10.58 ± 3.56	0.645
Creatinine (mg/dL)	0.74 ± 0.16	0.76 ± 0.17	0.73 ± 0.15	0.342
hs-CRP (mg/dL)	0.16 ± 0.30	0.17 ± 0.34	0.15 ± 0.19	0.810
eGFR (mL/min/1.73 m^2^)	104.09 ± 18.60	100.39 ± 15.81	107.89 ± 20.63	0.085
Glucose (mg/dL)	97.32 ± 10.70	93.89 ± 9.13	100.83 ± 11.16	0.005
T3 (ng/mL)	1.07 ± 0.16	1.04 ± 0.17	1.10 ± 0.15	0.156
Free T4 (ng/dL)	0.87 ± 0.11	0.89 ± 0.11	0.85 ± 0.11	0.126
TSH (μIU/mL)	2.19 ± 1.28	2.23 ± 1.13	2.16 ± 1.44	0.822

^1^ All variables were presented as mean ± standard deviation. ^2^ Scaled scores were used. Abbreviations: ALT, alanine transaminase; AST, aspartate aminotransferase; BDI, Beck Depression Inventory; BUN, blood urea nitrogen; hs-CRP, high sensitivity C-reactive protein; eGFR, estimated glomerular filtration rate; P300-A, amplitude of positive event-related potentials at 300 ms; P300-L, latency of positive event-related potentials at 300 ms; PSQI, Pittsburg Sleep Quality Index; PSS, Perceived Stress Score; STAI, State-Trait Anxiety Inventory; T3, triiodothyronine; T4, thyroxine; TSH, thyroid-stimulating hormone; WMS-III, Wechsler Memory Scale, 3rd edition.

## Supplementary Materials

The following are available online at https://www.mdpi.com/2072-6643/12/5/1362/s1, Table S1. Descriptions of the Cambridge Neuropsychological Test Automated Battery (CANTAB) tasks used in the current study; Table S2. The Cambridge Neuropsychological Test Automated Battery (CANTAB) outcome measure characteristics; Table S3. Reference values of self-report instrument; Table S4. Reference values of biochemistry markers.

## References

[1] Barnes D.E., Yaffe K. (2011). The projected effect of risk factor reduction on Alzheimer’s disease prevalence. Lancet Neurol..

[2] Deary I.J., Corley J., Gow A.J., Harris S.E., Houlihan L.M., Marioni R.E., Penke L., Rafnsson S.B., Starr J.M. (2009). Age-associated cognitive decline. Br. Med. Bull..

[3] Salthouse T.A. (2009). When does age-related cognitive decline begin?. Neurobiol. Aging.

[4] Singh-Manoux A., Kivimaki M., Glymour M.M., Elbaz A., Berr C., Ebmeier K.P., Ferrie J.E., Dugravot A. (2012). Timing of onset of cognitive decline: Results from Whitehall II prospective cohort study. BMJ.

[5] Karlamangla A.S., Lachman M.E., Han W., Huang M., Greendale G.A. (2017). Evidence for Cognitive Aging in Midlife Women: Study of Women’s Health Across the Nation. PLoS ONE.

[6] Walker K.A., Gottesman R.F., Wu A., Knopman D.S., Gross A.L., Mosley T.H., Selvin E., Windham B.G. (2019). Systemic inflammation during midlife and cognitive change over 20 years: The ARIC Study. Neurology.

[7] Echouffo-Tcheugui J.B., Conner S.C., Himali J.J., Maillard P., DeCarli C.S., Beiser A.S., Vasan R.S., Seshadri S. (2018). Circulating cortisol and cognitive and structural brain measures: The Framingham Heart Study. Neurology.

[8] Goodnite P.M. (2014). Stress: A concept analysis. Nurs. Forum..

[9] McGrath J.E., Dunnette M.D. (1976). Stress and behavior in organizations. Handbook of Industrial and Organizational Psychology.

[10] AbuAlRub R.F. (2004). Job stress, job performance, and social support among hospital nurses. J. Nurs. Scholarsh..

[11] Gandi J.C., Wai P.S., Karick H., Dagona Z.K. (2011). The role of stress and level of burnout in job performance among nurses. Ment. Health Fam. Med..

[12] Lue B.H., Chen H.J., Wang C.W., Cheng Y., Chen M.C. (2010). Stress, personal characteristics and burnout among first postgraduate year residents: A nationwide study in Taiwan. Med. Teach..

[13] Sandström A., Peterson J., Sandstrom E., Lundberg M., Nystrom I.L., Nyberg L., Olsson T. (2011). Cognitive deficits in relation to personality type and hypothalamic-pituitary-adrenal (HPA) axis dysfunction in women with stress-related exhaustion. Scand. J. Psychol..

[14] Wade A.T., Elias M.F., Murphy K.J. (2019). Adherence to a Mediterranean diet is associated with cognitive function in an older non-Mediterranean sample: Findings from the Maine-Syracuse Longitudinal Study. Nutr. Neurosci..

[15] Nooyens A.C., Milder I.E., van Gelder B.M., Bueno-de-Mesquita H.B., van Boxtel M.P., Verschuren W.M. (2015). Diet and cognitive decline at middle age: The role of antioxidants. Br. J. Nutr..

[16] Durga J., van Boxtel M.P., Schouten E.G., Kok F.J., Jolles J., Katan M.B., Verhoef P. (2007). Effect of 3-year folic acid supplementation on cognitive function in older adults in the FACIT trial: A randomised, double blind, controlled trial. Lancet.

[17] Morris M.C., Evans D.A., Tangney C.C., Bienias J.L., Schneider J.A., Wilson R.S., Scherr P.A. (2006). Dietary copper and high saturated and trans fat intakes associated with cognitive decline. Arch. Neurol..

[18] Wouters-Wesseling W., Wagenaar L.W., Rozendaal M., Deijen J.B., de Groot L.C., Bindels J.G., van Staveren W.A. (2005). Effect of an enriched drink on cognitive function in frail elderly persons. J. Gerontol. A Biol. Sci. Med. Sci..

[19] Denis I., Potier B., Heberden C., Vancassel S. (2015). Omega-3 polyunsaturated fatty acids and brain aging. Curr. Opin. Clin. Nutr. Metab. Care.

[20] Stough C., Downey L., Silber B., Lloyd J., Kure C., Wesnes K., Camfield D. (2012). The effects of 90-day supplementation with the omega-3 essential fatty acid docosahexaenoic acid (DHA) on cognitive function and visual acuity in a healthy aging population. Neurobiol. Aging.

[21] Stonehouse W., Conlon C.A., Podd J., Hill S.R., Minihane A.M., Haskell C., Kennedy D. (2013). DHA supplementation improved both memory and reaction time in healthy young adults: A randomized controlled trial. Am. J. Clin. Nutr..

[22] Jakobsen L.H., Kondrup J., Zellner M., Tetens I., Roth E. (2011). Effect of a high protein meat diet on muscle and cognitive functions: A randomised controlled dietary intervention trial in healthy men. Clin. Nutr..

[23] Markus C.R., Olivier B., de Haan E.H. (2002). Whey protein rich in alpha-lactalbumin increases the ratio of plasma tryptophan to the sum of the other large neutral amino acids and improves cognitive performance in stress-vulnerable subjects. Am. J. Clin. Nutr..

[24] Nagai H., Harada M., Nakagawa M., Tanaka T., Gunadi B., Setiabudi M.L., Uktolseja J.L., Miyata Y. (1996). Effects of chicken extract on the recovery from fatigue caused by mental workload. Appl. Hum. Sci..

[25] Azhar Z.M., Syedsahiljamalulail S. (2003). Effect of taking chicken essence on stress and cognition of human volunteers. Malays. J. Nutr..

[26] Azhar Z.M., Zubaidah J.O., Norjan K.O.N. (2008). Effect of taking chicken essence on cognitive functioning of normal stressed human volunteers. Malays. J. Med. Health Sci..

[27] Chan L., Wang H.M., Chen K.Y., Lin Y.C., Wu P.J., Hsieh W.L., Chen Y.R., Liu C.P., Tsai H.Y., Chen Y.R. (2016). Effectiveness of Essence of Chicken in Improving Cognitive Function in Young People Under Work-Related Stress: A Randomized Double-Blind Trial. Medicine.

[28] (2015). 28-Day Repeated Dose of Oral Toxicity Study in Rats—Chicken Meat Ingredient/CE Protein Powder (Unpublished Report).

[29] (2016). 28-Day Repeated Dose of Oral Toxicity Study in Rats—Chicken meat ingredient (Unpublished Report).

[30] (2019). A Human Tolerance, Safety, and Quality of Life Study on a Protein-Peptide Extract Health Supplement (Unpublished Report).

[31] Tsai S.F., Chang C.Y., Yong S.M., Lim A.L., Nakao Y., Chen S.J., Kuo Y.M. (2018). A Hydrolyzed Chicken Extract CMI-168 Enhances Learning and Memory in Middle-Aged Mice. Nutrients.

[32] Chou M.Y., Chen Y.J., Lin L.H., Nakao Y., Lim A.L., Wang M.F., Yong S.M. (2019). Protective Effects of Hydrolyzed Chicken Extract (Probeptigen(R)/Cmi-168) on Memory Retention and Brain Oxidative Stress in Senescence-Accelerated Mice. Nutrients.

[33] Azhar Z.M., Zubaidah J.O., Norjan K.O.N., Zhuang C.Y., Tsang F. (2013). A pilot placebo-controlled, double-blind, and randomized study on the cognition-enhancing benefits of a proprietary chicken meat ingredient in healthy subjects. Nutr. J..

[34] Ni Y., Ni L., Ma L., Wang Z., Zhao Y., Hu L., Zheng L., Fu Z. (2020). Neuroprotective Effects of ProBeptigen/CMI-168 on Aging-Induced Cognitive Decline and Neuroinflammation in Mice: A Comparison with Essence of Chicken.

[35] Cambridge Cognition CANTAB® (2016). Cognitive Assessment Software.

[36] Cohen S., Kamarck T., Mermelstein R. (1983). A global measure of perceived stress. J. Health Soc. Behav..

[37] Beck A.T., Steer R.A., Brown G.K. (1996). Manual for the Beck Depression Inventory-II.

[38] Spielberger C.D., Gorsuch R.L., Lushene R., Vagg P.R., Jacobs G.A. (1983). Manual for the State-Trait. Anxiety Inventory (STAI).

[39] Buysse D.J., Reynolds C.F., Monk T.H., Berman S.R., Kupfer D.J. (1989). The Pittsburgh Sleep Quality Index: A new instrument for psychiatric practice and research. Psychiatry Res..

[40] Faden A.I., Knoblach S.M., Movsesyan V.A., Cernak I. (2004). Novel small peptides with neuroprotective and nootropic properties. J. Alzheimers Dis..

[41] Tsuruoka N., Beppu Y., Koda H., Doe N., Watanabe H., Abe K. (2012). A DKP cyclo(L-Phe-L-Phe) found in chicken essence is a dual inhibitor of the serotonin transporter and acetylcholinesterase. PLoS ONE.

[42] Gudasheva T.A., Boyko S.S., Akparov V., Ostrovskaya R.U., Skoldinov S.P., Rozantsev G.G., Voronina T.A., Zherdev V.P., Seredenin S.B. (1996). Identification of a novel endogenous memory facilitating cyclic dipeptide cyclo-prolylglycine in rat brain. FEBS Lett..

[43] Prasad C. (1995). Bioactive cyclic dipeptides. Peptides.

[44] Tabassum S., Ahmad S., Madiha S., Khaliq S., Shahzad S., Batool Z., Haider S. (2017). Impact of oral supplementation of Glutamate and GABA on memory performance and neurochemical profile in hippocampus of rats. Pak. J. Pharm. Sci..

[45] Bohme G.A., Bon C., Lemaire M., Reibaud M., Piot O., Stutzmann J.M., Doble A., Blanchard J.C. (1993). Altered synaptic plasticity and memory formation in nitric oxide synthase inhibitor-treated rats. Proc. Natl. Acad. Sci. USA.

[46] Calabrese V., Mancuso C., Calvani M., Rizzarelli E., Butterfield D.A., Stella A.M. (2007). Nitric oxide in the central nervous system: Neuroprotection versus neurotoxicity. Nat. Rev. Neurosci..

[47] Cooke J.P., Dzau V.J. (1997). Nitric oxide synthase: Role in the genesis of vascular disease. Annu. Rev. Med..

[48] Colzato L.S., Jongkees B.J., Sellaro R., Hommel B. (2013). Working memory reloaded: Tyrosine repletes updating in the N-back task. Front. Behav. Neurosci..

[49] Shurtleff D., Thomas J.R., Schrot J., Kowalski K., Harford R. (1994). Tyrosine reverses a cold-induced working memory deficit in humans. Pharmacol. Biochem. Behav..

[50] Thomas J.R., Lockwood P.A., Singh A., Deuster P.A. (1999). Tyrosine improves working memory in a multitasking environment. Pharmacol. Biochem. Behav..

[51] Wechsler D. (1997). WMS-III: Wechsler Memory Scale Administration and Scoring Manual.

[52] The Psychological Corporation (1997). WAIS-III/WMS-III Technical Manual.

[53] Hua M.-S., Chang B.-S., Lin K.-N., Yang C.-M., Lu L.H.-J., Chen H.-Y. (2005). Wechsler Memory Scale- III (Chinese Version): Administration and Scoring Manual.

[54] Fray P.J., Robbins T.W., Sahakian B.J. (1996). Neuropsychiatric applications of CANTAB. Int. J. Geriatr. Psych..

[55] Cambridge Cognition CANTAB. http://www.cambridgecognition.com/technology.

[56] Strauss E., Sherman E.M.S., Spreen O. (2006). A Compendium of Neuropsychological Tests: Administration, Norms, and Commentary.

[57] Gerhart K.A., Weitzenkamp D.A., Kennedy P., Glass C.A., Charlifue S.W. (1999). Correlates of stress in long-term spinal cord injury. Spinal Cord..

[58] Lu M.-L., Che H., Chang S.W., Shen W.W. (2002). [Reliability and Validity of the Chinese Version of the Beck Depression Inventory-II]. Taiwan J. Psychiatry.

[59] Huang S.-L., Hsieh C.-L., Wu R.-M., Lu W.-S. (2017). Test-retest reliability and minimal detectable change of the Beck Depression Inventory and the Taiwan Geriatric Depression Scale in patients with Parkinson’s disease. PLoS ONE.

[60] Wang K.-C., Chung F.-C. (2016). An Investigation of Multidimensional Factorial Validity of the Chinese Version of State-Trait Anxiety Inventory. Psychol. Test..

[61] Tsai P.S., Wang S.Y., Wang M.Y., Su C.T., Yang T.T., Huang C.J., Fang S.C. (2005). Psychometric evaluation of the Chinese version of the Pittsburgh Sleep Quality Index (CPSQI) in primary insomnia and control subjects. Qual. Life Res..

[62] Squires N.K., Squires K.C., Hillyard S.A. (1975). Two varieties of long-latency positive waves evoked by unpredictable auditory stimuli in man. Electroencephalogr. Clin. Neurophysiol..

[63] Linden D.E. (2005). The p300: Where in the brain is it produced and what does it tell us?. Neuroscientist.

[64] Portin R., Kovala T., Polo-Kantola P., Revonsuo A., Muller K., Matikainen E. (2000). Does P3 reflect attentional or memory performances, or cognition more generally?. Scand. J. Psychol..

[65] Faul F., Erdfelder E., Buchner A., Lang A.G. (2009). Statistical power analyses using G*Power 3.1: Tests for correlation and regression analyses. Behav. Res. Methods.

[66] Liang K.-Y., Zeger S.L. (1986). Longitudinal data analysis using generalized linear models. Biometrika.

[67] Reder L.M., Wible C., Martin J. (1986). Differential memory changes with age: Exact retrieval versus plausible inference. J. Exp. Psychol. Learn. Mem. Cogn..

[68] Toh D.W.K., Wong C.H., Fam J., Kim J.E. (2019). Daily consumption of essence of chicken improves cognitive function: A systematically searched meta-analysis of randomized controlled trials. Nutr. Neurosci..

[69] Johnson W., Logie R.H., Brockmole J.R. (2010). Working memory tasks differ in factor structure across age cohorts: Implications for dedifferentiation. Intelligence.

[70] Baddeley A. (2000). The episodic buffer: A new component of working memory?. Trends Cogn. Sci..

[71] Salthouse T.A. (1996). The processing-speed theory of adult age differences in cognition. Psychol. Rev..

[72] Goodin D.S., Squires K.C., Starr A. (1978). Long latency event-related components of the auditory evoked potential in dementia. Brain.

[73] Parra M.A., Ascencio L.L., Urquina H.F., Manes F., Ibanez A.M. (2012). P300 and neuropsychological assessment in mild cognitive impairment and Alzheimer dementia. Front. Neurol..

[74] Kurihara H., Yao X.-S., Nagai H., Tsuruoka N., Shibata H., Kiso Y., Fukami H. (2006). Anti-Stress Effect of BRAND’S Essence of Chicken (BEC) on Plasma Glucose Levels in Mice Loaded with Restraint Stress. J. Health Sci..

[75] Terai K., Matsuo A., McGeer P.L. (1996). Enhancement of immunoreactivity for NF-kappa B in the hippocampal formation and cerebral cortex of Alzheimer’s disease. Brain Res..

[76] Wesnes K.A. (2014). Moving beyond the pros and cons of automating cognitive testing in pathological aging and dementia: The case for equal opportunity. Alzheimers Res. Ther..

[77] Leung D.Y., Lam T.-H., Chan S.S. (2010). Three versions of Perceived Stress Scale: Validation in a sample of Chinese cardiac patients who smoke. BMC Public Health.

