# Supplementary material for: Hydrolyzed Chicken Extract (ProBeptigen®) on Cognitive Function in Healthy Middle-Aged People: A Randomized Double-Blind Trial
Source: Nutrients. 2020 May 10;12(5):1362. doi: 10.3390/nu12051362 (PMC7284526; doi:10.3390/nu12051362)
Supplement: Supplementary file 1 [file nutrients-12-01362-s001.pdf]

Online supplemental material

The detailed information tasks of CANTAB selected in the study were adapted from the CANTAB manual as follows:

Table S1. Descriptions of CANTAB tasks used in the current study

**1. Reaction Time task (RTI)**<br>In this reaction time task, the participant holds down a button at the bottom of the screen untila yellow spot appears in one of five circles at the top of the screen. They must then release the button and touch inside the circle where the yellow spot appeared, as quickly as they can. Practice trials are included to familiarize participants with the task. The key outcome measure of the RTI is median five-choice reaction time (**RTIFMDRT**, lower scores indicating better performance).
**2. Paired Associates Learning (PAL)**<br>Boxes are displayed on the screen and open in turn to reveal a number of patterns. Participants are instructed to try and remember the location in which each pattern was shown. After all the boxes have opened, each pattern is then shown in the center of the screen in a randomized order, and the participant touches the box in which the pattern was located. If an error is made, all the patterns are re-presented to remind the participant of their locations. As the test progresses so the stages become more difficult as the number of patterns to be remembered increases. Three key outcome measures were used:<br>• Total errors adjusted (2-8 shapes, **PALTEA28**, lower scores indicating better performance) : The number of times the subject chose the incorrect box for a stimulus on assessed problems, plus an adjustment for the estimated number of errors they would have made on any problems, attempts and recalls they did not reach (up to and including the eight-shape stage). This measure allows you to compare performance on errors made across all subjects regardless of those who terminated early versus those who completed the eight-shape stage of the task.<br>• First attempt memory score (2-8 shapes, **PALFAMS28**, higher scores indicating better performance): The number of times a participant chose the correct box on their first attempt when recalling the pattern locations. Calculated across all assessed trials up to and including the eight-shape stage.<br>• Total errors adjusted (12 shapes, **PALTEA12**, lower scores indicating better performance): The number of times the participant chose the incorrect box for a stimulus on assessed problems, where the number of shapes was equal to 12, plus an adjustment for the estimated number of errors they would have made on any other 12 pattern problems, attempts and recalls they did not reach.
**3. Delayed Matching to Sample (DMS)**<br>A complex pattern is shown on the screen in a red box. Four similar patterns will then appear on the screen in white boxes below the red box. The participant is required to touch the pattern that exactly matches the target pattern in the red box. Sometimes the pattern in the red box will stay on the screen, and other times it will be covered up, so the participant must always try to remember it. At times, there will be a slightly longer wait before the patterns appear in the white boxes below the red box. The key outcome measure is percent correct (**DMSPC**, higher scores indicating better performance).
**4. Pattern Recognition Memory (PRM)**<br>A series of 18 patterns appear, one at a time, on the screen. These patterns are designed so that they cannot be easily given verbal labels. In the immediate recognition phase, the participant chooses which of two patterns they have already seen before. This is then replaced with a new set of patterns to be remembered. The participant is required to

complete the delayed recall phase after a delay of 20 minutes. Two key outcome measures were used:

- Percent Correct Immediate (**PRMPCI**, higher scores indicating better performance): The number of correct patterns selected by the participant in the immediate forced-choice condition, expressed as a percentage.
- Percent Correct Delayed (**PRMPCD**, higher scores indicating better performance): The number of correct patterns selected by the participant in the delayed forced-choice condition, expressed as a percentage.

5. Spatial Working Memory (SWM)

This task assesses the ability of the participant to retain spatial information and manipulate it in working memory. It is a self-ordered task that also gives a measure of strategy (an aspect of executive function). A number of colored boxes are presented on the screen, and the computer hides a token in these boxes one at a time. The participant is instructed to touch the boxes in turn to search for the token that has been hidden. When a token is found it should be placed in a home area on the right side of the screen. The participant then searches for more tokens until the same number of tokens as the number of colored boxes has been found. The key task instruction is that the computer will never hide a token in the same colored box twice in the same problem. As the test progresses, so it becomes more difficult as the number of boxes increases. Three key outcome measures were used:

- Between Errors (4-8 boxes, **SWMBE468**, lower scores indicating better performance): The number of times the participant incorrectly revisits a box in which a token has previously been found. Calculated across all assessed four, six and eight token trials.
- Between Errors (12 boxes, **SWMBE12**, lower scores indicating better performance): The number of times the participant incorrectly revisits a box in which a token has previously been found. Calculated across assessed twelve token trials only.
- Strategy (6-8 boxes, **SWMS**, lower scores indicating better performance): The number of times a participant begins a new search pattern from the same box they started with previously. If they always begin a search from the same starting point, we infer that the participant is employing a planned strategy for finding the tokens. Therefore, a low score indicates high strategy use (1 = they always begin the search from the same box), a high score indicates that they are beginning their searches from many different boxes. Calculated across all assessed six and eight token trials.

6. Rapid Visual Information Processing (RVP)

RVP is a sensitive measure of sustained attention, outputting measures of response accuracy, target sensitivity and reaction times. For the RVP task, single digits appear in a pseudo-random order at a rate of 100 digits per minute in a box in the centre of the screen. Participants must detect a series of 3-digit target sequences (e.g. 3-5-7; 2-4-6; 4-6-8) and respond by touching the button at the bottom of the screen when they see the final number of the sequence. Nine target sequences appear every minute. Three key outcome measures were used:

- Probability of a Hit (**RVPPH**, higher scores indicating better performance): The number of target sequences during assessed blocks that were correctly responded to within the time allowed, divided by the number of target sequences during assessed blocks.
- Median Response Latency (**RVPMDL**, lower scores indicating better performance): The median response latency on trials where the participant responded correctly.
- A Prime (**RVPA**, higher scores indicating better performance): A′ (A prime) is the signal detection measure of a participant's sensitivity to the target sequence (string of three numbers), regardless of response tendency (the expected range is 0.00 to 1.00; bad to good). In essence, this metric is a measure of how good the participant is at detecting target sequences.

7. **Memory Composite (CANTABCOM, higher scores indicating better performance)** In addition to the individual test endpoints, an overall memory composite score was also created. This was generated using z-scores from the following CANTAB measures: PAL Total Errors Adjusted (2-8 shapes) (**PALTEA28**), **SWM** Between Errors (4-8 boxes) (**SWMBE468**), **DMS** Percent Correct (**DMSPC**) and **PRM** Percent Correct Delayed (**PRMPCD**). All z-scores were based on the baseline mean and standard deviation, i.e. ((time point score – baseline mean) / baseline standard deviation (SD)) across the pooled dataset (placebo and treatment group).

Table S2. CANTAB outcome measure characteristics

**Outcome**	**Direction**	**Form**	**Range**
PAL total errors adjusted (**PALTEA28**)	Lower is better	Discrete ordinal	0-70
RTI median five-choice reaction time (**RTIFMDRT**)	Lower is better	Continuous	100-5100
PAL first attempt memory score (**PALFAMS28**)	Higher is better	Discrete ordinal	0-20
PAL total errors 12 shapes adjusted (**PALTEA12**)	Lower is better	Discrete ordinal	0-48
DMS percent correct **(DMSPC**)	Higher is better	Discrete ordinal	0-100
PRM percent correct immediate (**PRMPCI**)	Higher is better	Discrete ordinal	0-100
SWM between errors (**SWMBE**)	Lower is better	Discrete ordinal	0-153
SWM strategy (**SWMS**)	Lower is better	Discrete ordinal	2-14
RVP probability of a hit (**RVPPH**)	Higher is better	Continuous	0-1
RVP median latency (**RVPMDL**)	Lower is better	Continuous	100-1900
RVP A′ prime (**RVPA**)	Higher is better	Discrete ordinal	0-1
PRM percent correct delayed (**PRMPCD**)	Higher is better	Discrete ordinal	0-100
CANTAB memory composite score **(CANTABCOM)**	Higher is better	Continuous	Not available

Table S3. Reference values of self-report instrument

**Instruments**	**Direction**	**Cut-off**	**Range**
Perceived Stress Scale **(PSS)**	Lower is better	Not available	0-56
Beck Depression Inventory-second edition **(BDI-II)**	Lower is better	≥ 13 mild depression	0-63
State-Trait Anxiety Inventory **(STAI)** state anxiety	Lower is better	Not available	20-80
State-Trait Anxiety Inventory **(STAI)** trait anxiety	Lower is better	Not available	20-80
Pittsburg Sleep Quality Index **(PSQI)**	Lower is better	≥ 5 poor sleep quality	0-27

Table S4. Reference values of biochemistry markers†

**Biomarkers**	**Normal range/ cut-off value**
ALT (IU/L)	5-40 IU/L

AST (IU/L)	5-40 IU/L
BUN (mg/dL)	6-20 mg/dL
Cortisol (μg/dL)	4.30-22.40 μg/dL
Creatinine (mg/dL)	0.40-1.30 mg/dL
hs-CRP (mg/dL)	>0.3mg/dL indicating high risk
eGFR (mL/min/1.73 m^2)	>60 mL/min/1.73 m^2
Glucose (mg/dL)	70-100 mg/dL
T3 (ng/mL)	0.87-1.78 ng/mL
Free T4 (ng/dL)	0.58-1.64 ng/dL
TSH (μIU/mL)	0.27-4.20 μIU/mL
†The reference values were used in the institution that the study was conducted.	
